# Distinct Cell-specific Roles of NOX2 and MyD88 in Epileptogenesis

**DOI:** 10.3389/fcell.2022.926776

**Published:** 2022-07-04

**Authors:** Cayo Almeida, Renan Paschoalino Pongilio, Marília Inês Móvio, Guilherme Shigueto Vilar Higa, Rodrigo Ribeiro Resende, Jianxiong Jiang, Erika Reime Kinjo, Alexandre Hiroaki Kihara

**Affiliations:** ^1^ Laboratório de Neurogenética, Universidade Federal do ABC, São Bernardo do Campo, Brazil; ^2^ Laboratório de Sinalização Celular e Nanobiotecnologia, Departamento de Bioquímica e Imunologia, Universidade Federal de Minas Gerais, Belo Horizonte, Brazil; ^3^ Department of Pharmaceutical Sciences, College of Pharmacy, University of Tennessee Health Science Center, Memphis, TN, United States

**Keywords:** epilepsy, neuroinflammatory diseases, temporal lobe, single-cell analysis, cre recombinase, adeno-associated virus, toll-like receptors, transgenic mice

## Abstract

It is well established that temporal lobe epilepsy (TLE) is often related to oxidative stress and neuroinflammation. Both processes subserve alterations observed in epileptogenesis and ultimately involve distinct classes of cells, including astrocytes, microglia, and specific neural subtypes. For this reason, molecules associated with oxidative stress response and neuroinflammation have been proposed as potential targets for therapeutic strategies. However, these molecules can participate in distinct intracellular pathways depending on the cell type. To illustrate this, we reviewed the potential role of nicotinamide adenine dinucleotide phosphate (NADPH) oxidase 2 (NOX2) and myeloid differentiation primary response 88 (MyD88) in astrocytes, microglia, and neurons in epileptogenesis. Furthermore, we presented approaches to study genes in different cells, employing single-cell RNA-sequencing (scRNAseq) transcriptomic analyses, transgenic technologies and viral serotypes carrying vectors with specific promoters. We discussed the importance of identifying particular roles of molecules depending on the cell type, endowing more effective therapeutic strategies to treat TLE.

## Introduction

Temporal lobe epilepsy (TLE) is the most prevalent form of the disease, corresponding to ∼40% of the cases, of which one-third are refractory to the current pharmacological treatments (Guedes et al., 2006; Organization, W. H, 2019; Falco-Walter, 2020). It is already established that epileptogenesis is an ongoing process even after the first unprovoked seizure, since rearrangements of the neural networks constantly occur (Kinjo et al., 2016; Kinjo et al., 2018; Royero et al., 2020). This is due to stress factors such as free radicals’ production and neuroinflammation, and it contributes to the progression of epilepsy (Pitkänen et al., 2015; Jiang et al., 2019; Borowicz-Reutt and Czuczwar, 2020).

Free radicals, e.g., reactive oxygen species (ROS), play a physiological role in processes such as synaptic plasticity (Beckhauser et al., 2016). They also participate in the pathological aspects of TLE and several brain disorders, such as Alzheimer’s and Parkinson’s diseases, impairing neurotransmission and favoring lipid peroxidation of the neural membrane (Puttachary et al., 2015; Borowicz-Reutt and Czuczwar, 2020). Besides the contribution of ROS in epileptogenesis, damage-associated molecular patterns (DAMPs) interact with Toll-like receptors (TLRs) dependent on myeloid differentiation primary response 88 (MyD88) for glial cell activation, promoting the release of interleukin 1β (IL-1β) and other proinflammatory molecules (Paschon et al., 2016). The following glial activation and the ongoing communication between astrocytes and microglia can be of most importance in deciding the neuroinflammation fate of the tissue in a pathology (Paschon et al., 2016; Kinoshita and Koyama, 2021).

Indeed, genes related to oxidative stress and neuroinflammation play different roles depending on the cell type in epileptogenesis. In this context, we approached the role of nicotinamide adenine dinucleotide phosphate (NADPH) oxidase 2 (NOX2), one of the main sources of ROS triggered by prolonged seizures (Shekh-Ahmad et al., 2019), and MyD88 in epileptogenesis. Furthermore, we discussed the employment of single-cell RNA-sequencing (scRNAseq) transcriptomic analyses, transgenic technologies, and viral serotypes carrying vectors with specific promoters to unravel the role of genes related to oxidative stress and neuroinflammation in epilepsy.

## Distinct Roles of NOX2 in Microglia and Neurons

Microglial cells are known as the primary immune cells of the brain. As resident myeloid cells in the brain, they scour the environment removing cellular debris and releasing cytokines and free radicals such as ROS (Rettenbeck et al., 2015; Kinoshita and Koyama, 2021). Therefore, microglia plays a dual role in epileptogenesis, from distinct microglial phenotypes in sclerotic regions of the epileptic brain (Luo et al., 2016; Morin-Brureau et al., 2018) to promoting pro- and/or anti-epileptic activity (Kinoshita and Koyama, 2021).

Several studies demonstrated that *status epilepticus* (SE) elevates the level of ROS produced by microglia hours after its induction in animals (Dal-Pizzol et al., 2000; Sleven et al., 2006; Ryan et al., 2014). It has also been identified in the hippocampus of patients with refractory epilepsy (Shekh-Ahmad et al., 2019). The time course of ROS level alteration and the early microglial response may be associated with neural injury in the hippocampus following an insult that promotes epileptogenesis (Rettenbeck et al., 2015). Although other isoforms of the NOX family exist, NOX2 is of particular interest given its prominent expression by microglia. NOX2 is one of the main sources of ROS produced and released in epileptogenesis (Mcelroy et al., 2017; Shekh-Ahmad et al., 2019). In microglia, the GTPase Rab27 induces the movement of NOX2 complex to the plasma membrane (Ejlerskov et al., 2012). Nevertheless, in other cell types, Ras GTPase-activating-like protein (IQGAP1) is crucial for NOX2’s movement towards the cell surface (Ikeda et al., 2005). The inhibition of NOX2 or its genetic ablation in mice reduces microglial activity and neuroinflammation (Guemez-Gamboa et al., 2011; Wang et al., 2013; Zhang et al., 2017; Chandran et al., 2018). This data suggests that ROS modulates the inflammatory response and microglial activation in epileptogenesis (Chhor et al., 2013; Shekh-Ahmad et al., 2019).

**FIGURE 1:**
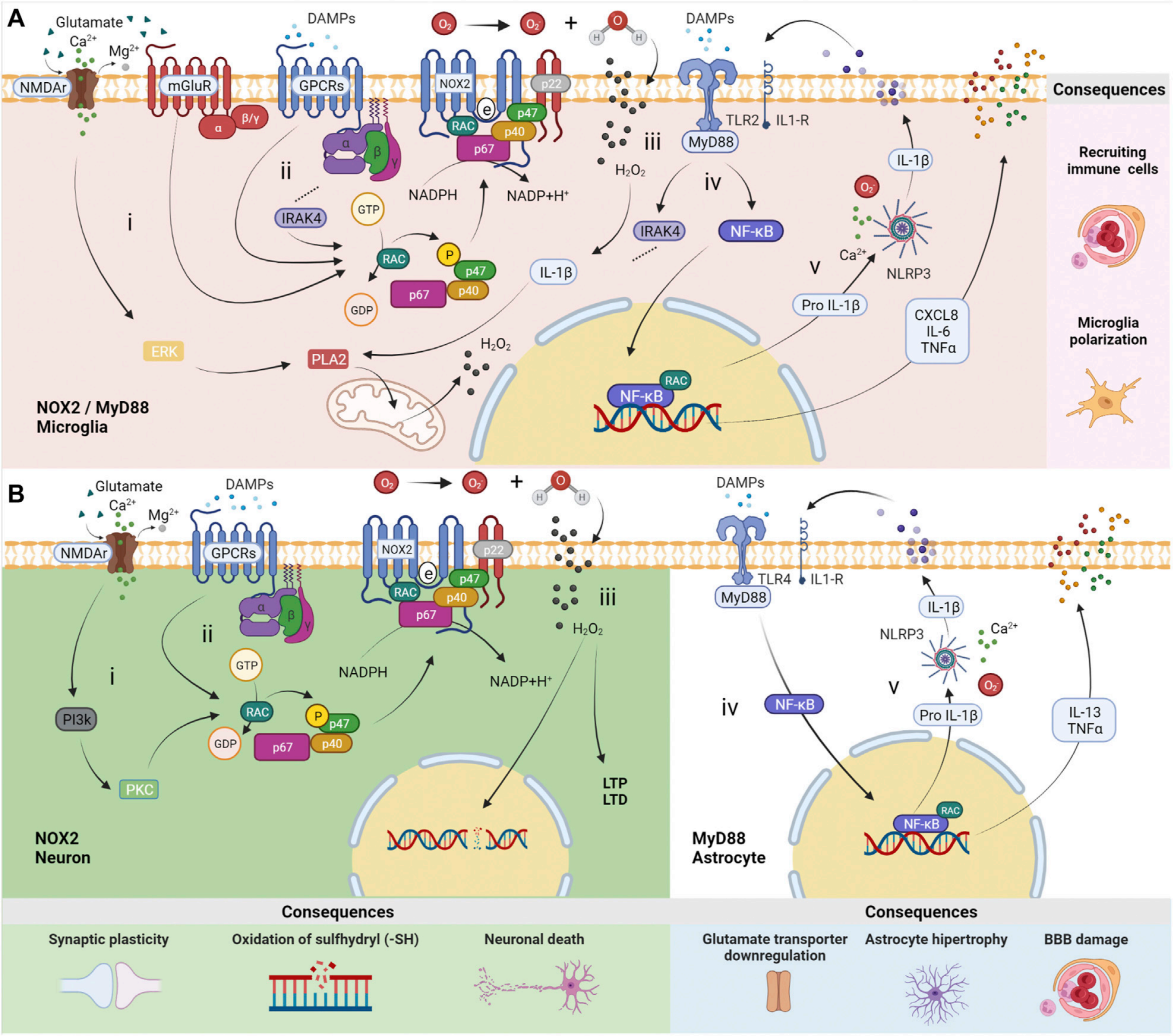
Distinct roles of MyD88 and NOX2 in neurons, astrocytes and microglia. **(A)** In microglia, (i) N-methyl-D-aspartate receptors (NMDAr) promote a calcium inflow, which leads to the production of mitochondrial reactive oxygen species (ROS) *via* ERK-PLA2. The activation of metabotropic glutamatergic receptors from group II (mGluR3) and group III (mGluR4, 6, 7, 8) induces NOX2 activation throughout G_i_/G_o_ alpha subunit. (ii) NOX2 can be activated by receptors of damage-associated molecular patterns (DAMPs) G-protein-dependent (GPCRs), i.e., N-formyl peptide receptors (FPRs) and P2Y receptors (P2YRs) *via* RAC, followed by phosphorylation of p47^phox^ (p47) and migration to the membrane along with p67^phox^ (p67) and p40^phox^ (p40), resulting in ROS production. (iii) H_2_O_2_ produced from hydrogen and O_2_ can cross membranes to participate in biochemistry reactions. (iv) Stimulation by DAMPs or cytokines throughout Toll-like Receptor 2 (TLR2) and interleukin-1 receptor (IL-1R), activates an intracellular signaling pathway involving the protein adaptor myeloid differentiation primary response 88 (MyD88). Specific consequences occur in microglia, such as NOX2 activation via IRAK4-RAC pathways. (v) NF-κB/RAC transcription factors promote the expression of chemokines CXCL8, IL-6, TNFα, and Pro IL-1β. The latter is transformed into mature IL-1β by the NLRP3 inflammasome dependent on calcium and ROS. The consequences are immune cell recruiting and microglia polarization. **(B)** Activation of NOX2 in neurons occurs (i) by NMDAr (containing subunit GluN2B) *via* PI3K-PKC and/or (ii) by DAMPs receptors (FPRs and P2YRs). (iii) H_2_O_2_ produced from hydrogen and O_2_ induces long-term potentiation (LTP) and long-term depression (LTD.) in physiological situations. However, a high concentration of ROS, e.g., in *status epilepticus* (SE), results in neural DNA damage by oxidation of sulfhydryl compounds (–SH) and cell death due to influx of H_2_O_2_ to the nucleus. In astrocytes, (iv) MyD88 activation by TLR4 is followed by NF-κB/RAC transcription factors assembly, (v) promoting the expression of IL-6, TNFα, and Pro IL-1β. The latter is transformed into mature IL-1β by the NLRP3 inflammasome dependent on calcium and ROS. The consequences include ions and neurotransmitters unbalance, astrocytic hypertrophy, and blood-brain barrier (BBB) changes. The illustrations were obtained using BioRender software.

NOX2 catalyzes molecular oxygen into its oxidative form in microglia and neurons. Its activation is modulated by N-methyl-D-aspartate receptors (NMDAr) and metabotropic glutamatergic receptors of group II (mGluR3) and group III (mGluR4, 6, 7, 8), specifically (Haslund-Vinding et al., 2017). Besides that, DAMPs receptors coupled to G protein, i.e., N- formyl peptide receptors (FPRs) and P2Y receptors (P2YRs), which, upon stimulation, promote phosphorylation via RAC of cytosolic p47^phox^, leading to its conformational change. Alongside p67^phox^ and p40^phox^, this protein migrates to the membrane. In their interaction with NOX2 and p22^phox^, electrons are transferred from the substrate NADPH to molecular oxygen, resulting in superoxide production (Vermot et al., 2021). Additionally, hydrogen peroxide (H_2_O_2_) is suitable for triggering the phospholipase A2 (PLA2) pathway through signal-regulated kinase (ERK) and IL-1β production, which targets the IL-1β receptor and MyD88 pathway (Figures 1Ai,ii,iii) (Beckhauser et al., 2016).

The localization of NOX2 in the postsynaptic region is convenient for its role in synaptic plasticity (Bedard and Krause, 2007; Brennan et al., 2009). ROS physiologically acts as a second messenger in the nervous system, mostly supporting synaptic long-term potentiation (LTP) (Hidalgo and Arias-Cavieres, 2016) or long-term depression (LTD.) (Francis-Oliveira et al., 2018). In the hippocampus, for instance, ROS activates protein kinases that are essential for neural plasticity. Upon stimulation, NMDAr (containing the subunit GluN2B) promotes a calcium inflow into synapses, which leads to the production of ROS via Phosphoinositide 3-Kinase (PI3K)—Protein Kinase C (PKC), H_2_O_2_, and neural nitric oxide synthase (nNOS) (Beckhauser et al., 2016). Once produced, H_2_O_2_ can pass through the plasma membrane to interact with pre- and postsynaptic proteins, modulating the synaptic transmission (Quinn and Gauss, 2004). However, it can also induce damage on neuronal DNA, including chromosomal alterations, breakage of the DNA column and, in the absence of catalyst, oxidation of sulfhydryl compounds (–SH), leading to cell death in pathological conditions (Figures 1Bi,ii,iii) (Beckhauser et al., 2016).

## Distinct Roles of MyD88 in Microglia and Astrocytes

TLRs 2, 4, and 9 are expressed in astrocytes, while TLRs 1-9 are expressed in microglia. However, in neuroinflammation, the innate immune response mediated by microglia and astrocytes depends largely on TLR2 and TLR4, respectively, and IL-1R in both cell types. Most TLRs are coupled to the MyD88 adapter, the exception being TLR3, which is activated by double-strand DNA triggering the TRIF pathway. It is also important to mention that TLR4 can be activated by lipopolysaccharide to signal both pathways MyD88 and TRIF (Paschon et al., 2016; Fiebich et al., 2018).

These receptors can be activated by DAMPs, such as High Mobility Group Box 1 (HMGB-1), as observed in epilepsy, and beta-amyloid plaques and alpha-synuclein, in Alzheimer’s and Parkinson’s disease, respectively (Bernaus et al., 2020). These DAMPs can interact with TLR receptors located in astrocytes and microglia membranes. TLR activation leads both cells to secrete cytokines and inflammatory molecules that activate NADPH, which aids NOX2 and cyclooxygenase 2 (COX2) activation in microglia (Meng and Yao, 2020). The secretion of cytokines promotes autocrine and paracrine signaling, leading astrocytes and microglia activation into a vicious circle (Vezzani et al., 2013; Bernaus et al., 2020).

In the literature, it is regularly reported the expression of correspondent TLRs by astrocytes and microglia, however, the release of specific cytokines by these cells depends on the stimulus or pathology. For instance, after seizure activity, the microglia of patients with TLE release IL-1β, CXCL8, IL-6 and TNFα (Morin-Brureau et al., 2018). On the other hand, in an LPS-induced Parkinson’s model, the up-regulation of IFN-γ, IL-1β, IL-1R, IL-16, IL-17 levels in microglia have been reported. Therefore, it can be inferred that cytokines’ release by microglia depends on many factors, although using the same intracellular signaling pathway, the NF-κB and NLRP3 inflammasome (Chien et al., 2016). When immunologically stimulated by TNFα or IL-1β, post-mortem or biopsy human astrocytes and cell-line NT2 astrocytes release cytokines IL-1β, TNFα, and IL-13. However, post-mortem or biopsy human astrocytes also produced IL-2, IL-7, TNFβ, and IF N-γ (Figures 1iv,v) (Burkert et al., 2012).

Microglia activation by TLRs and MyD88 modulate distinct epileptogenesis features. In this cell type, MyD88 signaling promotes cytokine secretion and induces apoptosis of neighboring cells. Microglia potentializes the immune response by recruiting peripheral immune system cells, increasing the inflammatory environment (Meng and Yao, 2020). TLR2 signalling activates IL-1 Receptor-Associated Kinase 4 (IRAK4) in the MyD88-dependent axis. Once activated, IRAK4 phosphorylates p47phox on several residues to activate NOX2 (Pacquelet et al., 2007). Besides that, microglia disrupts gamma-aminobutyric acid (GABA) signaling and regulates the neural expression of NR1 and NR2b subunits of NMDA receptor. These processes result in high excitability of neurons which, in turn, contributes to epileptogenesis (Vezzani et al., 2013; Liu et al., 2018; Xueying Li et al., 2021).

On the other hand, MyD88 signaling modulates the astroglial tissue-maintenance functions, such as ions and neurotransmitters homeostasis (Li et al., 2020; Vezzani et al., 2013). In neuroinflammation, astroglial activation through the MyD88 pathway creates a harmful environment to the neural tissue, characterized by an astrocytic hypertrophic state, which impairs the blood-brain barrier and neural communication maintenance. (Vezzani et al., 2013; Liu et al., 2018; Xueying Li et al., 2021). Astrocytic activation can occur by MyD88-dependent TLR4 via the extracellular signal-related kinase (ERK) pathway, and promotes excitatory synapse development, resulting in increased seizure susceptibility (Henneberger and Steinhauser, 2016; Shen et al., 2016). In this context, the increased expression of TLR4 in astrocytes, but not in microglia, suggests TLR4’s role in seizure frequency in human epilepsy (Pernhorst et al., 2013).

The crosstalk involving the MyD88 pathway in microglia and astrocytes seems crucial in triggering inflammation in the central nervous system (CNS), given that the absence of microglia *in vitro* and *in vivo* leads to a failure of TLR4 activation in astrocytes, consequently reducing the inflammatory response (Barbierato et al., 2013; Liddelow et al., 2017). In epileptogenesis, neuroinflammation contributes to the pathological synaptic plasticity and dendrite growth by activating IL-1R/TLR receptors, transcription factor NF-κB, and cytokines effects (Vezzani et al., 2013; Meng and Yao, 2020).

Therefore, anti-inflammatory therapies and pharmacological drugs that influence the signaling pathway involving TLR4/MyD88/NF-κB/NLRP3/IL-1β, such as baicalin (TLR4 inhibitor) and agmatine (TLR4 down regulator), also reduce the frequency, duration and intensity of seizures (Lun Li et al., 2021; Yang et al., 2021). Furthermore, MyD88 inhibition reduces phosphorylation and expression of NMDAr, modulates proinflammatory microglia, and increases the expression of glutamate receptors in astrocytes (Wang et al., 2017; Liu et al., 2018). These mechanisms support the important role of MyD88 in neuroinflammation associated with epilepsy; in this sense, approaches that directly target MyD88 inhibition demonstrate positive effects in epilepsy. In summary, MyD88 and NOX2 are essential players in epileptogenesis, regulating the neuroinflammatory response in a cell-specific manner, promoting distinct outcomes.

## Discussion

Considering that specific cell types have distinct impacts on epilepsy, technologies that allow screening, such as scRNAseq, genetically modified animals, and cell-targeted gene control provide valuable approaches to study epilepsy. New therapeutic strategies are necessary since around one-third of patients with TLE are refractory to clinically available drug treatments.

**FIGURE 2:**
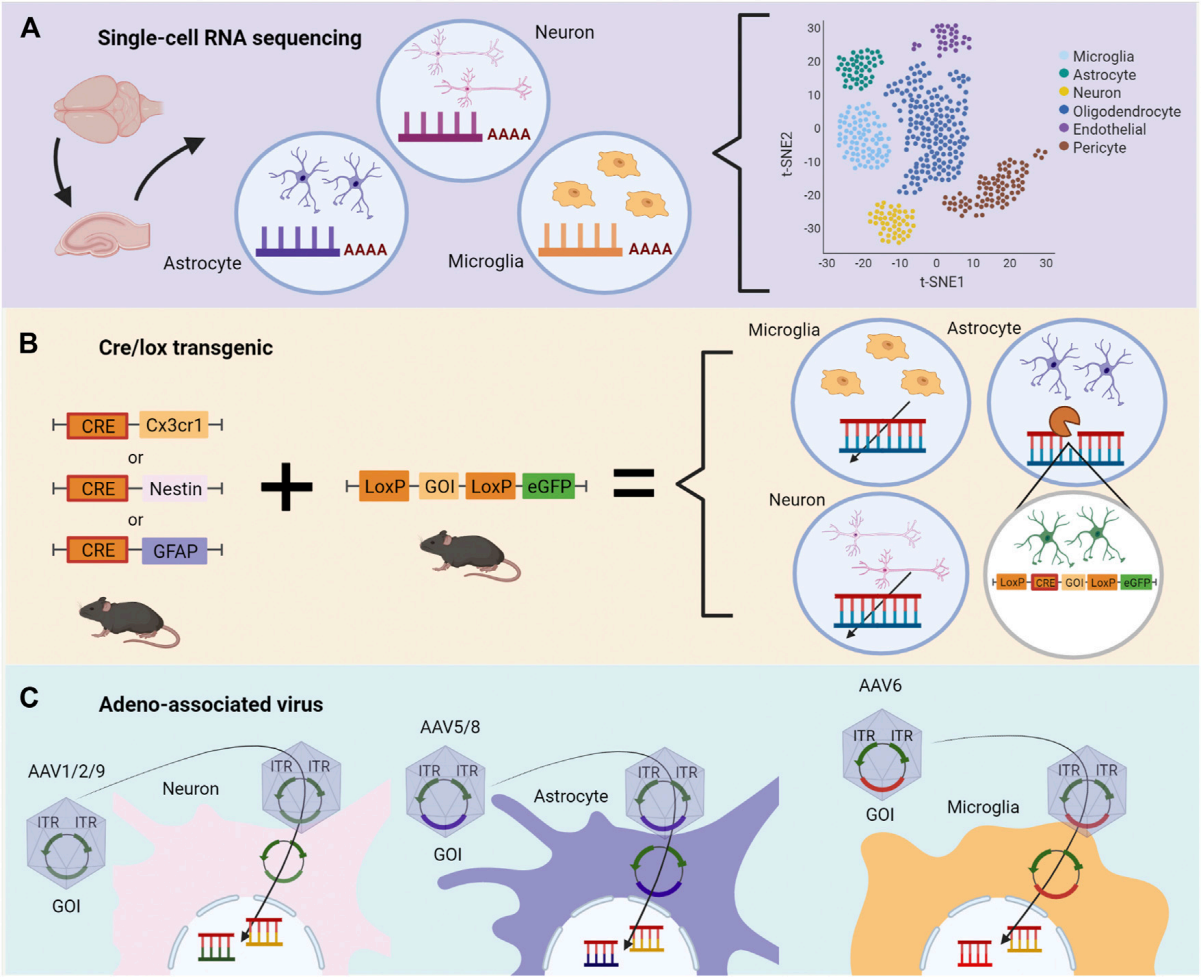
Methodological strategies to assess specific roles of genes during epileptogenesis depending on the cell type. **(A)** Single-cell RNA sequencing (scRNA-seq) of the temporal lobe or hippocampus permits the evaluation of changes in transcriptomics triggered by epileptogenesis. Distinct cell types are separately analyzed, and a complete RNA profile is obtained. Changes in transcriptomics are described according to correlation, heatmaps, and quantitative analysis. **(B)** Cre/lox transgenic technologies can be used to evaluate the role of genes in cell-type-specific during epileptogenesis *in vivo*. In the first generation, two distinct animals are bred, one containing the Cre enzyme, the other containing two LoxP sequences between the gene of interest (GOI) and a gene reporter, as eGFP. Littermates receive genetic material necessary for the Cre/flox system to modify, delete or change the GOI. **(C)** Adeno-associated virus (AAV) contains the GOI with inverted terminal repeats (ITR) and viral capsid necessary for cell-specific targeting. The genetic modification occurs by the transported plasmid carrying the GOI. The illustrations were obtained using BioRender software.

scRNAseq allows the assessment of the transcriptomic profile of cell-type-specific (Figure 2A). This technique offers insights into the mechanisms involved in epileptogenesis and characterizes the specific contribution of microglia, astrocytes, and neurons in epilepsy (Olsen and Baryawno, 2018; Cid et al., 2021). This approach investigates NOX2 and MyD88 transcriptomic pathways in distinct time points in epileptogenesis in animal models and surgically-extracted nervous tissues. Specific cell types are separately analyzed, and a complete RNA profile is obtained. For instance, RNAseq data of epileptic hippocampus demonstrated the possibility of investigating oxidative stress regulation and inflammatory responses, such as cytokines, cell migration, proliferation, and cell death, which involve TLR2, TLR3, TLR4, TLR7, and C1qa, inflammasome genes, and pyroptosis (Cid et al., 2021).

Besides mapping changes in the transcriptome in cell-type-specific, transgenic animals allow the control of gene expression in a cell-specific manner, such as in the Cre/flox system (Figure 2B). Cre-mice breed with LoxP (floxed) mice results in Cre-LoxP animals. In this condition, Cre enzyme promotes the inversion or deletion of the genes flanked by LoxP sequences, which can be replaced by a reporter gene such as eGFP (Kim et al., 2018). Bringing this approach into the context of specific targets in epilepsy, it is possible to track the downstream effects of MyD88 and NOX2 loss-of-function in cell-type-specific. Indeed, the cell-type-specific gene ablation combined with pharmacological approaches has been demonstrated particularly useful in the determination of the roles of COX2 (Serrano et al., 2011) and prostaglandin receptor EP2 (Nagib et al., 2020; Sluter et al., 2021; Varvel et al., 2021) in animal models of SE.

For example, to test the downstream effects of MyD88 loss in microglia, a transgenic mice model was established to target microglia chemokine receptors (Cx3cr1) to knockout the MyD88 gene. The authors showed a relationship between neuron maturation and loss of MyD88 in microglia cells, which disrupts the reward-related-memory formation in morphine addiction (Rivera et al., 2019). On the other hand, the Nestin-Cre model directly induces gene modification into neurons and microglia, which showed that the presence of MyD88 in both cell types is not necessary to respond to a nocive stimulus (Braun et al., 2012). Specific neuron modifications are usually achieved with the Synapsin I Cre model (Mcnair et al., 2020), which is capable of modifying differentiated neurons. Regarding astrocytes, although the most used model is the GFAP-Cre, it also promotes changes in non-specific targets; S100b and GLAST are alternatives pointed out to decrease the off-target effects (Guttenplan and Liddelow, 2019; Mcnair et al., 2020). Also, the generation of brain-specific Mn-SOD-deficient mice (brain-Sod22/) to study ROS in the hypoxia model (Sasaki et al., 2011) and neurodegenerative diseases (Marecki et al., 2014; Park et al., 2022) demonstrates the application of this technology.

In addition to transgenic animal models, genetic modifications through viral vectors have also opened opportunities for studying TLE (Figure 2C). Amongst these, adeno-associated virus (AAV) stands out given that its variations have distinct tropism for cell-type-specific. Also, AAV vectors have been used for gene therapy applications due to their safety and the ability to infect dividing and non-dividing cells, performing a long-term transgene expression profile (Daya and Berns, 2008). For instance, AAV can be applied to increase CCL2, aiming to rescue the expression of cytokine levels (CCL2, IL-1β), since these cytokines were described as reduced by other therapies (Wu et al., 2019).

AAV allows control of the gene expression by inserting the gene of interest (GOI), or a sequence that generates a short hairpin RNA (shRNA) to target the GOI, in the host DNA. This modification promotes overexpression or knockdown of the GOI in the host cell (Daya and Berns, 2008). Several serotypes have been identified in AAV, increasing the probability of transfection of cell-type-specific. For CNS, serotype 2 (AAV2) is the most adopted virus despite the variability of transfection efficiency regarding the cell type, which is overcome by using pseudotyping AAVs by recombining new capsids from different serotypes (Fakhiri and Grimm, 2021). Neurons and astrocytes are efficiently transfected by AAV, both *in vivo* and *in vitro*. Also, vectors have been tested for transfection efficiency and transgene expression, demonstrating that AAV5 (Griffin et al., 2019) and AAV8 (Aschauer et al., 2013) are potent viral vectors to transfect astrocytes, while AAV2/1 (Hammond et al., 2017) and AAV9 (Aschauer et al., 2013) are best suitable for neurons. Microglia are the most refractile cells in the nervous system to AAV transfection, but several improvements have been made. For instance, a modified rAAV6 combined with specific microglial promoters succeeded in transfecting these cells *in vitro* and *in vivo* (Rosario et al., 2016; Su et al., 2016). Therefore, by employing specific serotypes, it is possible to overexpress or knockdown the expression of genes implied in the epileptogenesis process, such as NOX2 and MyD88, in cell-type-specific.

Cell-type-specific approaches grant benefits in research and scientific study development. However, the lack of reproducibility and technical difficulties might be the reasons for possibly delivering biased results, such as in the RNA-seq case (Whitley et al., 2016). In the Cre/lox system, specific tissue promoters can be expressed in undesired tissues and at uncommon times (Sauer, 1998). At last, their use for in-human treatment may not be indicated, such as in the case of AAV therapies, which hold genotoxicity potential (Colella et al., 2018). Epilepsy has already demonstrated to be multifactorial, therefore a single treatment based on monotherapy will probably not be efficient, especially in refractory TLE (Vezzani et al., 2013; Organization, W. H, 2019; Falco-Walter, 2020).

In summary, cell-type-specific approaches should be applied to investigate mechanisms related to brain disorders. This strategy promotes a better understanding of genes involved in epileptogenesis, bringing new insights into treatments and therapies for TLE (Couvillion et al., 2009).
